# Bridging Cancer and COVID‐19: The Complex Interplay of ACE2 and TMPRSS2

**DOI:** 10.1002/cam4.70829

**Published:** 2025-03-27

**Authors:** Xuerui Tang, Liuzhi Lu, Xiaoping Li, Panpan Huang

**Affiliations:** ^1^ School of Basic Medicine Gannan Medical University Ganzhou Jiangxi China; ^2^ Clinical Laboratory Tongxiang First People's Hospital Zhejiang China

**Keywords:** angiotensin‐converting enzyme 2, cancer, coronavirus disease 2019, immune, severe acute respiratory syndrome coronavirus 2, transmembrane serine protease 2

## Abstract

The coronavirus disease 2019 (COVID‐19) pandemic presents heightened risks for cancer patients, who are more susceptible to severe acute respiratory syndrome coronavirus 2 (SARS‐CoV‐2) infection and severe outcomes due to immunosuppression from both the malignancy and anticancer therapies. This review investigates the dual roles of angiotensin‐converting enzyme 2 (ACE2) and transmembrane serine protease 2 (TMPRSS2) in SARS‐CoV‐2 infection among cancer patients. ACE2, the vital entry receptor for SARS‐CoV‐2, is overexpressed in certain tumors such as colon adenocarcinoma, renal carcinomas, pancreatic adenocarcinoma, and lung adenocarcinoma, potentially increasing viral susceptibility. Paradoxically, ACE2 also exhibits tumor‐suppressive properties by inhibiting angiogenesis and modulating the tumor microenvironment, leading to improved patient prognoses in some cancers like breast cancer. TMPRSS2, essential for viral entry, shows decreased expression in several tumors but acts as a prognostic biomarker in prostate and lung cancers. This review illustrates the complexity of therapeutically targeting ACE2 and TMPRSS2 due to their contrasting roles in cancer progression and viral entry. We analyze the expression levels of ACE2 and TMPRSS2 in relation to immune cell infiltration and patient outcomes, and propose personalized therapeutic strategies. Furthermore, we underscore the necessity for multidisciplinary approaches, integrating antiviral treatments with cancer therapies and tailoring interventions based on individual molecular profiles. This approach to personalized medicine seeks to enhance treatment results and better manage cancer patients who have contracted SARS‐CoV‐2.

## Graphical abstract

ACE2 and TMPRSS2 not only mediate SARS‐CoV‐2 infection may also be involved in cancer progression (such as affecting tumor proliferation, matastasis or immune infiltration), and they can be used as targets to treat cancer patients infected with SARS‐CoV‐2.

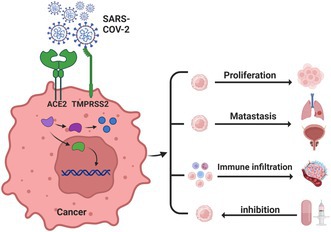

## 1 Introduction

The global health systems have faced unparalleled challenges due to the COVID‐19 pandemic, which is caused by the severe acute respiratory SARS‐CoV‐2. According to WHO data, as of January 19, 2025, the cumulative number of confirmed COVID‐19 patients reported worldwide has reached 778 million, and the number of deaths reported is as many as 7.1 million, indicating that the epidemic has a wide and sustained impact on the world. The database from CCC19 (https://ccc19.org/) showed that 242 (26%) of the 928 cancer patients from the United States, Canada, and Spain were severe patients. Patients with malignancies represent a particularly vulnerable population, as emerging evidence suggests that they are at an increased risk of contracting SARS‐CoV‐2 infection and experiencing severe disease outcomes [1, 2]. Cancer patients are more susceptible to SARS‐CoV‐2 due to several factors, including the malignancy itself and the immunosuppressive effects of anticancer therapies such as chemotherapy, radiotherapy, and targeted treatments [2, 3]. Cancer‐associated inflammation may lead to an exaggerated cytokine response upon infection with SARS‐CoV‐2, potentially resulting in a cytokine storm and multiorgan failure [2, 4, 5].

In addition to the general risk factors, understanding the molecular mechanisms underlying the increased susceptibility of cancer patients to SARS‐CoV‐2 is crucial. ACE2 is pivotal in the pathogenesis of COVID‐19, serving as the indispensable entry receptor for SARS‐CoV‐2 [6, 7]. ACE2 is a transmembrane protein extensively expressed in various human tissues, including lungs, heart, kidneys, intestines, and other organs, which accounts for the broad tropism of the virus and the diverse clinical manifestations observed in infected individuals [6, 8]. Beyond its role as a viral receptor, ACE2 has significant physiological importance. It functions as an enzyme and modulates the renin–angiotensin system (RAS) by converting angiotensin II to angiotensin‐(1–7), thereby counteracting the harmful effects of angiotensin II, such as vasoconstriction, inflammation, and fibrosis [6, 9]. TMPRSS2 plays a pivotal role in the entry of SARS‐CoV‐2 into host cells by priming the viral spike (S) protein, a crucial step for viral infectivity [10, 11]. TMPRSS2 is extensively present in different tissues, including the lungs, gastrointestinal tract, and prostate [3, 10].

ACE2 and TMPRSS2 expression in cancerous tissues might influence susceptibility to SARS‐CoV‐2 infection and the severity of COVID‐19 outcomes [9, 12]. For instance, elevated ACE2 and TMPRSS2 expression observed in lung adenocarcinoma (LUAD) patients may partially explain their increased vulnerability to SARS‐CoV‐2 infection [4]. On the other hand, genetic and epigenetic modifications common in cancers can impact the expression and function of ACE2 and TMPRSS2. Dysregulation of the ACE2/Angiotensin‐(1–7)/Mas receptor axis has been implicated in various cancers, influencing processes such as cell proliferation, angiogenesis, and metastasis [13]. In the context of cancer, especially prostate cancer (PCa), TMPRSS2 is of particular interest due to its androgen‐regulated expression and involvement in oncogenic processes [10, 14]. The interplay between ACE2, TMPRSS2, and immune mechanisms adds another layer of complexity. Altered immune environments in cancer patients, characterized by dysregulated cytokine production and immune cell functions, can influence both cancer progression and COVID‐19 severity [15].

In conclusion, elucidating the molecular mechanisms in comorbid cancer patients is vital for improving clinical outcomes. Therefore, a detailed exploration of ACE2 and TMPRSS2 in the context of SARS‐CoV‐2 infection is necessary to understand their impact on cancer patients.

## 2 Expression and Alterations of ACE2 in Malignancies

### 2.1 Overexpression of ACE2 in Specific Tumors

**FIGURE 1:**
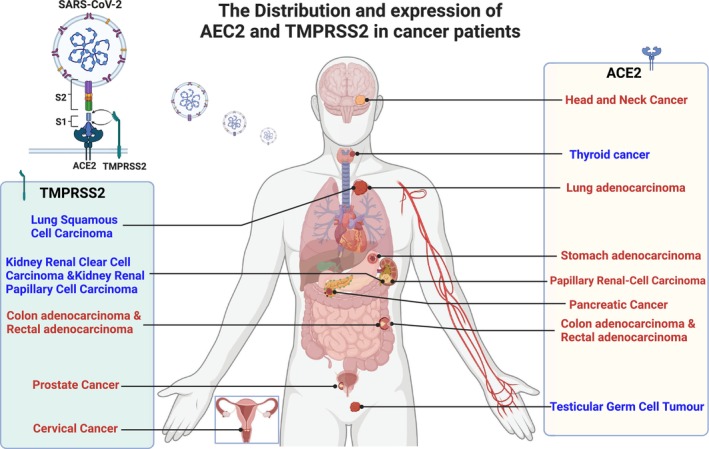
The distribution and expression of ACE2 and TMPRSS2 in cancer patients. ACE2 is highly expressed in colon adenocarcinoma (COAD), papillary renal cell carcinoma (KIRP), pancreatic adenocarcinoma (PAAD), rectum adenocarcinoma (READ), stomach adenocarcinoma (STAD), lung adenocarcinoma (LUAD), and head and neck cancer(HNSC).while the expression in testicular germ cell tumors (TGCT) and thyroid carcinoma (THCA) was lower than that in normal tissues. TMPRSS2 was highly expressed in cervical cancer, colon adenocarcinoma (COAD), prostate adenocarcinoma(PRAD), rectal adenocarcinoma (READ). The expression in kidney renal clear cell carcinoma (KIRC), kidney renal papillary cell carcinoma (KIRP) and lung squamous cell carcinoma (LUSC) was lower than that in normal tissues. Figure created with BioRender.com.

A pan‐cancer analysis revealed that ACE2 is overexpressed in various tumors, including colon adenocarcinoma (COAD), kidney renal papillary cell carcinoma (KIRP), pancreatic adenocarcinoma (PAAD), rectum adenocarcinoma (READ), stomach adenocarcinoma (STAD), and LUAD [8]. The aberrant expression of ACE2 in these tumors raises concerns regarding the susceptibility of cancer patients to SARS‐CoV‐2 infection, as higher ACE2 levels could facilitate viral entry into host cells (Figure 1).

In breast cancer (bc), ACE2 has been shown to inhibit angiogenesis, a key process in tumor progression and metastasis, by suppressing the vascular endothelial growth factor A (VEGFA)/VEGFR2/ERK signaling pathway [16]. This suggests that while ACE2 may promote viral entry, it also possesses tumor‐suppressive properties that could influence cancer progression. Notably, patients with higher ACE2 expression in bc exhibited longer relapse‐free survival, indicating a potential protective role of ACE2 against aggressive tumor behavior [16, 17].

Furthermore, in kidney cancers such as clear cell renal cell carcinoma (ccRCC) and KIRP, ACE2 expression has been correlated with various pathological features and immune infiltration [18, 19, 20]. Downregulation of ACE2 in these cancers has been associated with advanced disease stages and poorer prognoses, suggesting that ACE2 may serve as a prognostic biomarker [20]. The relationship between ACE2 expression and immune cell infiltration further underscores its potential role in modulating the tumor microenvironment and influencing patient outcomes.

In summary, the overexpression of ACE2 in specific tumors presents a dual‐edged sword. While it may enhance susceptibility to SARS‐CoV‐2 infection, it also appears to play a role in inhibiting tumor progression and influencing patient prognosis.

### 2.2 ACE2 Downstream Signaling Pathways Effects in Cancer Patients With COVID‐19 Infection

**FIGURE 2:**
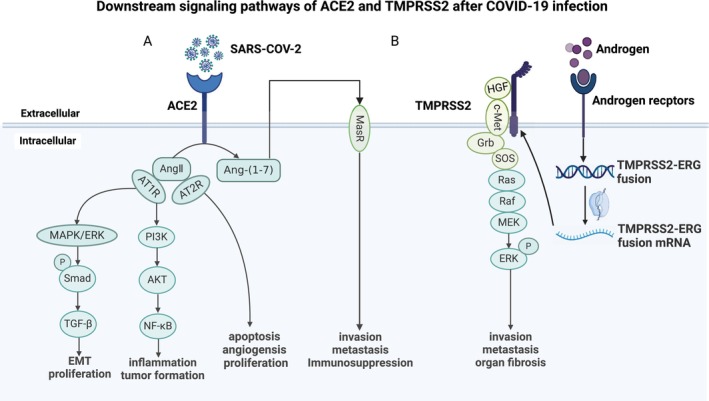
Downstream signaling pathways of ACE2 and TMPRRS2 after COVID‐19 infection. ACE2 catalyzed the conversion of AngII into Ang‐(1–7). The binding of AngII to AT1R and AT2R, respectively, mediates the signaling pathways such as MAPK/ERK signaling pathways and PI3K/AKT signaling pathways, leading to inflammatory response, tumor formation, EMT and proliferation, angiogenesis, and apoptosis. Ang‐(1–7) induces changes in tumor invasion and metastasis, immunosuppression through MASR‐mediated signaling pathways; **(B)** Androgen regulates TMPRSS2 transcription process. TMPRSS2 and HGF bind to activate the Ras/Raf/MAPK pathway through binding molecules and c‐Met to induce tumor proliferation and invasion. Figure created with BioRender.com.

ACE2 dysregulation has been shown to be one of the important factors affecting the pathophysiological process and severity of SARS‐CoV‐2 infection in cancer patients [1]. ACE2 is homologous to angiotensin‐converting enzyme (ACE) and is a negative regulator of the RAS [21]. It is known that the endocytosis of SARS‐CoV‐2 reduces the expression of ACE2 on the cell membrane, which may lead to the accumulation of Ang II and the weakening of the protection capacity of the ACE2/Ang‐(1–7)/MASR axis, so that Ang II overacts on angiotensin II type 1 receptor (AT1R) and angiotensin II type 2 receptor (AT2R). It causes immune dysfunction and pathophysiological changes [22]. According to the database, AT1R is lowly expressed in COAD, kidney chromophobe (KICH), breast invasive carcinoma (BRCA), and LUAD (GEPIA (cancer‐pku.cn)). It is involved in tumor growth, invasion, and metastasis, oxidation, proinflammatory response, and angiogenesis, and other pathophysiological reactions [23, 24]. AT1R activates the PI3K/AKT/NF‐κB signaling pathway to induce bc cells to upregulate VEGF to promote angiogenesis, cell proliferation, and inspire the production of adhesion molecules (such as E‐selectin) and epithelial–mesenchymal transition (EMT) to depolarize tumor cells in order to invade and migrate to adjacent tissues, affecting the progression and survival of tumor patients [25]. It is also possible that AT1Rs may bind to Ang II to trigger other intracellular biochemical reactions, such as Smad2 and Smad3 phosphorylation by activating MAPK/extracellular signal‐regulated kinase (ERK) signaling pathways. Phosphorylated Smad2 and Smad3 interact with Smad4 to form a complex that promotes TGF‐β transcription [26]. TGF‐β/Smad3 and TGF‐β/p38/MAPK signaling pathways are involved in the production of extracellular matrix (ECM) and induce EMT formation. Cell proliferation, metastasis, and apoptosis lead to increased tumor incidence and accelerated progression [27]. Upregulation of AT1R inhibits the proliferation of LUAD cells through the PI3K/AKT3 pathway, accelerates apoptosis, forms an antitumor immune microenvironment, and improves the prognosis of patients [28]. According to the database, AT2R is lowly expressed in lung cancer, endometrial cancer, pancreatic cancer, and bladder cancer (GEPIA (cancer‐pku.cn)). In addition to human cell proliferation and differentiation, AT2R is associated with antiinflammatory responses, antifibrosis, apoptosis, and other pathophysiological processes [29]. Induction of AT2R overexpression in bladder cancer resulted in increased expression levels of caspase‐3, caspase‐8, and p38, decreased expression levels of p‐Erk and VEGF, promoted apoptosis, and inhibited tumor cell proliferation and angiogenesis [30]. In spite of this, AT2R is tumor type‐specific, and the specific mechanism is unknown. In general, targeting AT1R and AT2R may be a potential diagnostic indicator and treatment for cancer patients with RAS system disorders. There is still a need for further research on the special role of AT1R and AT2R in tumorigenesis and development, as well as factors affecting patient prognoses. Based on the above research, it is speculated that the RAS system needs to be a key point in the treatment of cancer pathophysiology and novel coronavirus fusion (Figure 2). ACE2 and TMPRSS2 are the entry points of SARS‐CoV‐2, and their downstream signaling pathways play an important role in the tumor microenvironment. PI3K/AKT signaling pathway changes the M2 polarization of tumor stem cells (TAM) and other pathways such as TAM autophagy, and participates in the biological behaviors of tumor cell proliferation, invasion, recruitment, and apoptosis [31]. In addition, TGF‐β plays multiple roles in the tumor microenvironment. It not only inhibits adaptive immunity by promoting the expansion of regulatory T cells (Treg) and inhibiting the function of effector T cells, but also regulates innate immunity by inhibiting the function of NK cells, macrophages, DCs, and granulocytes. This systemic immunosuppressive effect helps tumor cells evade immune surveillance. Neutralizing TGF‐β can enhance the antitumor immune response of CD8+ T cells and NK cells, and promote the recruitment and activation of neutrophils with antitumor phenotype [32, 33]. A potential solution to prevent complications from COVID‐19 is to regulate the RAS system, which has potential protective effects to prevent disease progression. This suggests that ACE2 could be a promising important therapeutic target for new coronary patients, as well as a theoretical basis for the development of other complications.

## 3 Expression and Alterations of TMPRSS2 in Malignancies

### 3.1 Expression and Clinical Outcomes of TMPRSS2 in Cancers

TMPRSS2 plays a crucial role in the entry of SARS‐CoV‐2 into host cells. Beyond its role in viral entry, TMPRSS2 is implicated in the biology of various cancers, particularly PCa. Djomkam et al. highlighted that TMPRSS2 is abundantly expressed in the prostate gland and is significantly involved in PCa biology [34]. Their commentary suggests that SARS‐CoV‐2's exploitation of TMPRSS2 may influence prostate carcinogenesis by modulating androgen receptor (AR) signaling, promoting TMPRSS2–ERG gene fusions, and inducing chronic inflammation—all factors associated with prostate tumorigenesis [34, 35] (Figure 1).

Liu et al. conducted a comprehensive multi‐omics analysis of TMPRSS2 expression and clinical outcomes across various cancers. Their research showed that TMPRSS2 expression is significantly reduced in numerous tumor tissues. Notably, the reduced TMPRSS2 levels were significantly linked with clinical outcomes in brain, blood, colorectal, breast, ovarian, lung, and soft tissue cancers [36]. Protein network analysis identified 27 protein partners of TMPRSS2, suggesting its involvement in regulating cancer progression. Additionally, a high level of TMPRSS2 was associated with immune cell infiltration, indicating its potential role in tumor immunity and as a therapeutic target during the COVID‐19 pandemic [36]. In LUAD, Liu et al. demonstrated that TMPRSS2 mRNA and protein expression are significantly reduced [37]. Lower TMPRSS2 expression and higher DNA methylation were linked to negative clinicopathological features and unfavorable outcomes in lung cancer patients. Notably, TMPRSS2 expression is correlated with immune cell infiltration, suggesting its role in modulating the tumor microenvironment. The study found that downregulation of TMPRSS2 was linked to increased proliferation, stemness, genomic instability, tumor progression, and worse survival in LUAD. In vitro and in vivo experiments confirmed that TMPRSS2 deficiency enhances tumor cell proliferation and invasion while modulating antitumor immunity. Importantly, TMPRSS2‐knockdown tumors exhibited increased sensitivity to programmed cell death protein 1 (PD‐1)/programmed cell death ligand‐1 (PD‐L1) inhibitors, suggesting therapeutic implications in immunotherapy [38]. Expanding on these findings, another study observed that elevated TMPRSS2 expression is significantly connected to immune infiltration in PCa and positively correlates with prognosis [37]. The reduced expression of TMPRSS2 in SARS‐CoV‐infected cells hints at the potential susceptibility of tumor tissues in COVID‐19 patients with PCa to SARS‐CoV‐2 infection, which may worsen prognosis. In kidney cancers, Tang et al. investigated the roles of ACE2 and TMPRSS2, given that the kidneys are targets for SARS‐CoV‐2 infection [19]. Their bioinformatics analyses indicated that downregulation of ACE2 and TMPRSS2 worsens the prognosis of kidney renal clear cell carcinoma (KIRC) and KIRP patients through metabolic pathways and immunoregulation. These findings underscore the importance of TMPRSS2 in kidney cancer prognosis, especially in the context of COVID‐19.

Collectively, these studies highlight the complex role of TMPRSS2 in cancer biology and its interaction with SARS‐CoV‐2 infection. TMPRSS2 expression varies across different cancer types, influencing tumor progression, immune infiltration, and patient prognosis. In some cancers, such as prostate and rectal adenocarcinomas, TMPRSS2 is overexpressed and associated with better immune responses, and correlates with poor outcomes and enhanced tumor aggressiveness.

### 3.2 TMPRSS2 Downstream Signaling Pathways Effects in Cancer Patients With SARS‐CoV‐2 Infection

According to the literature and database, TMPRSS2 promotes HGF and c‐Met receptor tyrosine kinase signal transduction. HGF and c‐Met complex‐mediated PCa cell invasion and metastasis‐related pathways [39, 40]. Binding of HGF to TMPRSS2 results in autophosphorylation of c‐Met. Activation of Ras/Raf/MAPK pathway and EMT by linker molecules confers cell migration and invasion characteristics, inhibits apoptosis and cell senescence, promotes immunosuppression, leads to organ fibrosis, and promotes PCa metastasis and invasion [39, 41]. Studies have found that AR inhibits c‐Met expression by inhibiting Sp1‐induced c‐Met gene transcription. In the preclinical model, the antitumor effect of the c‐Met inhibitor combined with androgen ablation therapy was greatly enhanced [42]. Therefore, the targeting of TMPRSS2‐mediated tumor downstream signaling pathways has therapeutic potential, but the underlying mechanisms remain to be determined.

In PCa, TMPRSS2 is directly regulated by AR transcription and regulated by androgen in vivo, which promotes tumor cell invasion and metastasis to distant organs and increases disease severity and infection risk [14]. Both malignant and nonmalignant prostate cells can experience TMPRSS2–ERG gene fusion as a result of androgen exposure [43]. TMPRSS2–ERG gene fusion is an important molecular marker of PCa, which can promote tumor cell proliferation and metastasis [44, 45]. The function and activity of ERG have been studied and related to cell migration, invasion, EMT, and metastasis, and some downstream targets have been reported, including Myc, Wnt/LEF1, and Notch signaling pathways [46, 47, 48]. ERG also cooperates with the PI3K‐AKT signaling pathway to regulate the progression of PCa [49]. The possible molecular relationship between fusion‐positive PCa patients and an increased risk of COVID‐19 infection could use TMPRSS2 as an attractive drug target [45]. The treatment of inhibiting TMPRSS2 may reduce the incidence or progression of metastasis in PCa patients with COVID‐19. However, these pathways associated with TMPRSS2 are currently limited to PCa and cannot fully reflect all tumors. Therefore, further study of the downstream signaling pathway of TMPRSS2 and its relationship with other tumors is crucial for understanding the pathogenesis of diseases and developing effective treatment methods (Figure 2).

The intracellular ERK/MEK pathway upregulates PD‐L1 in tumor cells in the tumor microenvironment, thereby activating the PD‐1/PD‐L1 pathway. The PD‐1/PD‐L1 pathway enhances tumor cell immune escape by increasing immunosuppressive cells and inhibiting tumor‐suppressing NK cells [50]. The expression level of TMPRSS2 is closely related to tumor immunity and prognosis. Therefore, understanding the expression of TMPRSS2 in tumor patients and its relationship with prognosis is helpful to elucidate the reasons why tumor patients are more prone to COVID‐19 infection, and to determine whether tumor immunotherapy affects their susceptibility.

## 4 Immune Correlations and Tumor Microenvironment

The interplay between ACE2, TMPRSS2, and immune cell infiltration is crucial for understanding cancer progression and susceptibility to SARS‐CoV‐2 infection [51]. Recent studies have shed light on how the expression of ACE2 and TMPRSS2 in tumor tissues influences the tumor microenvironment and impacts immune responses (Table 1).

**TABLE 1:** Expression of ACE2 and TMPRSS2 in tumor tissues in relation to immune cells and prognosis.

Cancer	Gene expression	Different immune cell levels	Prognosis	Reference
NSCLC	ACE2↑	CD8+ cytotoxic T lymphocytes and natural killer cells↑	No quiet clear	[52]
Luminal bc	ACE2↑	CD8+ T cells and neutrophils↓	Unfavorable prognosis	[17]
PCa	TMPRSS2↑	CD8+ T cells, dendritic cells, and macrophages↑	Favorable prognosis	[32]
Lung cancer	TMPRSS2↓	cytotoxic T cells↓	Unfavorable prognosis	[37]
bc	TMPRSS2↑	CD4+ T cells, neutrophils, and dendritic cells↑	Unfavorable prognosis	[53]

Hu et al. conducted an extensive analysis of ACE2 and TMPRSS2 expression across normal and tumor tissues, emphasizing their relationship with immune cell infiltration [52]. Notably, the infiltration of immune cells and the release of various cytokines were important factors contributing to cytokine storms observed in severe COVID‐19 cases. The altered expression of ACE2 and TMPRSS2 in cancer patients might influence immune cell behavior within the tumor microenvironment, potentially exacerbating the severity of COVID‐19 outcomes. In the context of non–small‐cell lung cancer (NSCLC), Mostafa et al. explored the expression of ACE2 in normal lung tissues and its relationship with immune cell infiltration [54]. The study found that patients with high ACE2 expression exhibited increased levels of CD8+ cytotoxic T lymphocytes and natural killer cells in normal lung tissues. However, the function of these immune cells might be impaired due to elevated expression of Thymocyte Selection‐Associated High Mobility Group Box Protein TOX and immune checkpoint molecules such as PD‐1, PD‐L1, CTLA‐4, and TIGIT [55] (Table 3). This immune‐tolerant state could contribute to an increased risk of severe COVID‐19 pneumonia in NSCLC patients, highlighting the complex role of ACE2 in immune regulation. Jiang et al. investigated the role of abnormal ACE2 expression in bc patients and its correlation with immune infiltration and prognosis. The study found that ACE2 expression was significantly decreased in bc tissues, except for the basal‐like subtype. In luminal bc, reduced ACE2 levels were associated with poorer prognosis and abnormal immune infiltration, particularly decreased CD8+ T cells and neutrophils [17].

Liu et al. investigated TMPRSS2 expression and its association with immune infiltration in various cancers through a multi‐omics approach [36]. Their findings indicated that high levels of TMPRSS2 were linked to increased infiltration of immune cells, such as lymphocytes and macrophages, in tumor tissues. This association suggests that TMPRSS2 plays a role in modulating the immune response within the tumor microenvironment [36]. In PCa, Luo et al. reported that elevated TMPRSS2 expression correlates with higher levels of infiltrating immune cells, including CD8+ T cells, dendritic cells, and macrophages [53]. Patients with higher TMPRSS2 expression demonstrated better overall survival rates, implying that TMPRSS2 may enhance immune‐mediated tumor suppression. This association underscores the potential of TMPRSS2 as a favorable prognostic biomarker in PCa. Similarly, in lung cancer, Liu et al. observed that decreased TMPRSS2 expression is linked to reduced immune cell infiltration, particularly of cytotoxic T cells [37]. This reduction may facilitate immune evasion by tumor cells, leading to disease progression and poorer clinical outcomes. The study suggests that TMPRSS2 downregulation may impair immune surveillance in lung cancer patients. Furthermore, Liu et al. performed an integrated analysis using single‐cell RNA sequencing data from COVID‐19 patients, LUAD, and small cell lung carcinoma patients to investigate preexisting vulnerability factors [59]. They identified that elevated TMPRSS2 levels in epithelial cells and more intense inflammatory reactions mediated by macrophages were shared pathological traits in both COVID‐19 and lung cancer. Additionally, they observed increased suppression of T‐cell responses and elevated fibrosis risk due to pathological fibroblasts. These preexisting conditions in lung cancer patients may result in heightened inflammation and diminish adaptive immune responses upon SARS‐CoV‐2 infection, highlighting the critical role of TMPRSS2 in immune modulation. Conversely, in bc, high TMPRSS2 expression was positively related to the infiltration of CD8+ T cells, CD4+ T cells, neutrophils, and dendritic cells. However, unlike in LUAD, increased TMPRSS2 expression in bc was associated with poor prognosis, particularly in patients with higher levels of CD4+ and CD8+ T cell infiltration [60]. This indicates that while TMPRSS2 may influence immune cell infiltration, its impact on patient outcomes varies between cancer types, potentially due to differences in tumor biology and immune contexture. The interaction between TMPRSS2 expression and immune responses is particularly relevant in the context of COVID‐19. Hu et al. proposed that altered expression of TMPRSS2 in cancer patients could influence cytokine production upon SARS‐CoV‐2 infection, potentially exacerbating the risk of a cytokine storm—a hyperinflammatory response leading to severe tissue damage and organ failure [52]. This diminished immune presence could impair antitumor immunity and increase vulnerability to SARS‐CoV‐2 infection, potentially worsening patient outcomes after infection.

The prognostic significance of immune infiltration in cancer patients becomes even more critical in the backdrop of the COVID‐19 pandemic. Latif et al. highlighted that patients with malignancies exhibit distinct immune dysfunctions, categorized broadly into “hot” (T‐cell‐inflamed) and “cold” (non‐T‐cell‐inflamed) tumors [15]. “Hot” tumors have a hyperactivated immune environment, potentially leading to excessive inflammatory responses upon SARS‐CoV‐2 infection, such as cytokine storms. In contrast, “cold” tumors display immune quiescence or senescence, which may impair effective immune responses against the virus. The altered immunological milieu in cancer patients, characterized by dysregulated cytokine profiles, can modulate the expression of ACE2 and TMPRSS2, further impacting the course of SARS‐CoV‐2 infection [15]. For instance, proinflammatory cytokines prevalent in “hot” tumors might upregulate ACE2 and TMPRSS2 expression, facilitating viral entry and propagation. This interplay suggests that immune infiltration not only affects cancer prognosis but also influences susceptibility and outcomes related to COVID‐19.

The intricate interplay between ACE2, TMPRSS2, and immune cell infiltration underscores the need for further research to elucidate their roles in cancer progression and susceptibility to SARS‐CoV‐2 infection. A deeper understanding of these mechanisms may facilitate the development of novel therapeutic strategies addressing both oncological challenges and infectious risks in cancer patients.

## 5 Potential Therapeutic Implications

### 5.1 ACE2 and TMPRSS2 as Therapeutic Targets

The expression levels and effects of ACE2 and TMPRSS2 were significantly different in different cancer types (Figure 1). ACE2 enhances the drug sensitivity of tumor cells through the Mas‐mediated signaling pathway, and may also inhibit the proliferation, metastasis, invasion, and angiogenesis of various tumor cells such as breast cancer, colon cancer, and lung cancer through other pathways [9]. TMPRSS2–ERG gene fusion may induce tumor invasion, metastasis, and angiogenesis in prostate cancer through gene mutation or immune regulation [61]. Therefore, ACE2 and TMPRSS2 can provide new therapeutic targets and therapeutic strategies for tumor therapy during the novel coronavirus epidemic. TMPRSS2 is essential for facilitating viral fusion with host cell membranes and subsequent entry [7, 10]. Inhibition of TMPRSS2 activity has emerged as a viable strategy to impede viral infection. Deng et al. demonstrated that camostat mesylate, a TMPRSS2 inhibitor, effectively blocked the cleavage of a pseudotyped SARS‐CoV‐2 spike protein without disrupting the interaction between TMPRSS2 and ACE2 [62]. At present, carmustine mesylate has been used in the treatment of oral squamous cell carcinoma and chronic pancreatitis [63]. Experiments have shown that quercetin and paclitaxel have effective inhibitory activity on extracellular and membrane‐bound TMPRSS2, and significantly inhibit the proliferation and cell cycle of bc cells, accompanied by changes in cell morphology [64].

In PCa, TMPRSS2 is regulated by the AR, which is activated by hormones like testosterone and dihydrotestosterone [14]. Mollica et al. discussed the dual role of TMPRSS2 in COVID‐19 and PCa, emphasizing that therapies targeting TMPRSS2 might confer benefits in both conditions. The aberrant regulation of TMPRSS2, particularly the TMPRSS2–ERG gene fusion, is associated with PCa development and progression. Androgen deprivation therapy (ADT) or AR antagonists can downregulate TMPRSS2 expression, potentially reducing both tumor growth and SARS‐CoV‐2 infectivity [14, 62]. In AR‐positive bc, proxalutamide as a novel potent AR antagonist has been approved for the treatment of PCa. Preclinical and phase I clinical trials have shown that probucol can effectively inhibit the growth of AR‐positive bc tumors [65]. For patients with mild‐to‐moderate COVID‐19, pramipramide has been shown to decrease ACE2 and TMPRSS2 expression in lung cancer cells, prevent SARS‐CoV‐2 from entering host cells, and facilitate faster virus clearance [66]. In addition, it can also inhibit the secretion of inflammatory factors such as TNF‐a and IFN‐γ caused by SARS‐CoV‐2 by regulating host immune and inflammation‐related signaling pathways and prevent the occurrence of cytokine storms, thereby generating positive clinical benefits [67]. In addition, hepatocyte growth factor activator inhibitor 2 (HAI‐2) can inhibit the activity of TMPRSS2, reduce the invasion and metastasis of PCa cells, and reduce SARS‐CoV‐2 infection [68, 69]. Meanwhile, this inhibition also attenuated SARS‐CoV‐2 spike‐mediated cellular entry, suggesting that TMPRSS2 inhibitors could potentially prevent COVID‐19 progression.

In LUAD patients, Tang et al. observed significantly increased ACE2 expression, which may contribute to their susceptibility to SARS‐CoV‐2 [4]. The study also identified miR‐432‐5p as a potential regulatory molecule targeting ACE2, proposing bexarotene as a drug that could modulate ACE2 expression and exert antiinflammatory effects [4]. Bexarotene's ability to influence ACE2 and TMPRSS2 expression offers a novel therapeutic avenue for treating COVID‐19 in lung cancer patients [21]. Spironolactone can reduce the amount of soluble ACE2 and antagonize TMPRSS2. Combinations of spironolactone with other inhibitors, such as DPP‐4, could prevent SARS‐CoV‐2 from entering cells and enhance antiinflammatory, antiproliferative, and antifibrotic effects, leading to improved clinical outcomes for COVID‐19 patients [70].

ACE2 is essential for maintaining healthy cardiopulmonary function [71]. Therefore, drugs that do not significantly affect the physiological activity of ACE2 are needed. Monoclonal antibodies (mAbs) are important biological therapeutic agents. Monoclonal antibodies against ACE2 have been used against SARS‐CoV‐2, which provides a promising therapeutic strategy for reducing SARS‐CoV‐2 infection [72]. For example, REGN‐COV2 (casirivimab and imdevimab) is a mixture of human Abs, casirivimab and imdevimab, targeting S protein RBD [73]. During the first half of 2021, Bamlanivimab and Etesevimab (Eli Lily) were among the top 10 best‐selling COVID‐19 drugs, along with REGN‐COV2. These treatments are approved for emergency use in adults with mild to moderate COVID‐19 [74]. In view of the above ACE2 effects and drug characteristics, we can rationally use ACE2 inhibitors for cancer patients with high expression of ACE2, while for patients with low expression of ACE2 or poor prognosis and unknown mechanisms such as KICH, TGCT, and THCA, ACE2 inhibitors should be used with caution.

ACE2 and TMPRSS2 are not only key molecules for SARS‐CoV‐2 to enter host cells, but also important auxiliary proteins that mediate other coronaviruses (such as SARS‐CoV) infection. Severe acute respiratory syndrome coronavirus (SARS‐CoV) and human coronavirus NL63 use ACE2 as an important receptor for entry into cells, causing zoonotic infection [75]. In the TMPRSS2 knockout mouse model, the airway inflammatory response in mice was significantly reduced. The transmission of viruses such as MERS‐CoV and SARS‐CoV in mice was significantly inhibited [37]. Therefore, exploring the mechanisms or therapeutic ideas involving ACE2 and TMPRSS2 can provide new insights into the fight against other coronaviruses. Targeting ACE2 and TMPRSS2 not only has important prospects as a therapeutic strategy against COVID‐19 and other coronaviruses, especially for cancer patients who may change the expression of these proteins due to malignant tumors or treatments. The dual role of TMPRSS2 in facilitating viral entry and influencing tumor progression makes it an attractive target for interventions that could benefit both cancer control and viral infection prevention [14, 62]. Similarly, careful modulation of ACE2 activity could reduce susceptibility to SARS‐CoV‐2 while maintaining essential physiological functions. However, altering ACE2 expression must be approached cautiously due to its vital physiological roles, including regulation of blood pressure and antiinflammatory effects in the renin–angiotensin system. Completely inhibiting ACE2 could have detrimental consequences, so strategies aiming for partial modulation or functional inhibition without affecting expression levels may be more appropriate (Table 2).

**TABLE 2:** Treatment of cancer patients infected with SARS‐CoV‐2 by targeting ACE2 and TMPRSS2.

Drugs	Object of action	Mode of action	Effect	Reference
Camostat mesilate	TMPRSS2	Inhibition of TMPRSS2 expression	Blocking the cleavage of pseudo‐SARS‐CoV‐2 spike protein; treatment of oral squamous cell carcinoma and chronic pancreatitis	[59]
Quercetin and paclitaxel	TMPRSS2	Inhibition of TMPRSS2 activity	Inhibit the proliferation and cell cycle of bc cells	[61]
Androgen deprivation therapy (ADT)	Androgen	Reduces androgen levels	Standard first‐line therapy for prostate cancer and significantly less likely to be infected with SARS‐CoV‐2 than patients not treated with ADT	[76]
Proxalutamide	Androgen receptor	Preventing androgen to bind to the nuclear androgen receptor	Inhibits bc tumor growth, prevents COVID‐19 invasion of host cells, and reduces secretion of inflammatory factors	[62, 63, 64]
Estrogens (e.g., estradiol)	Androgen receptor	Inhibition of TMPRSS2 expression	Proven to be effective and safe in the treatment of prostate cancer	[77]
Hepatocyte Growth Factor Activator Inhibitor 2 (HAI‐2)	TMPRSS2	Homologous inhibitor of TMPRSS2	Reduces prostate cancer cell invasion and metastasis and has been shown to reduce SARS‐CoV‐2 infection	[65, 66]
Bexarotene	ACE2 and TMPRSS2	Inhibition of ACE2 and TMPRSS2 expression	Regulates ACE2 expression and exerts antiinflammatory effects	[4, 21]
Combined use of spironolactone and DPP‐4	ACE2 and TMPRSS2	Reducing the amount of soluble ACE2 and antagonizing TMPRSS2	Blocking SARS‐CoV‐2 entry into cells and providing better antiinflammatory, antiproliferative and antifibrotic effects	[67]
Monoclonal antibodies (mAbs)	ACE2	Neutralization of ACE2	Inhibition of SARS‐CoV‐2 entry	[69]

**TABLE 3:** Potential links between ACE2 and TMPRSS2 and inhibitory checkpoint molecules.

Molecules	Effect	Potential links	Reference
PD‐1/PD‐L1	Immune checkpoint molecules, inhibit T cell activity, help tumors escape immune surveillance.	ACE2 may affect the expression and function of PD‐1/PD‐L1 by regulating the inflammatory response in the tumor microenvironment. Increasing the expression levels of ACE2 and TMPRSS2 can improve the efficacy of anti‐PD‐1 immunotherapy.	[56] [57] [58]
CTLA‐4	Immune checkpoint molecules, inhibit T cell activation, regulate immune tolerance	ACE2 may indirectly affect the expression of CTLA‐4 by regulating the differentiation and function of T cells.	[55]

### 5.2 Immunotherapy of Cancer Patients With COVID‐19

Therapies such as PD‐1/PD‐L1 blocking (restoring T cell ability in cancer and chronic viral infections) may enhance harmful hyperimmune responses in COVID‐19 patients, but they can provide much‐needed immune control of viral infections [15]. Pd‐L1‐blocking antibody (PD‐L1mAb) is crucial in immune regulation, as it effectively lowers inflammation by suppressing the production of ACE2, IL‐5, and granulocyte‐macrophage colony‐stimulating factor (GM‐CSF) [78]. Overexpression of ACE2 inhibited immunosuppression and angiogenesis induced by M2‐like tumor‐associated macrophages (TAM) and showed a decreased CCR5 + PD‐L1+ immunosuppressive phenotype. It enhanced the tumor suppressive effect of anti‐PD‐L1 therapy and inhibited hepatocellular carcinoma (HCC) progression [79]. ACE2 recognizes immunothermal tumors in bc, and its enzyme product Ang‐1‐7 makes bc sensitive to chemotherapy and immunotherapy by remodeling TME [76]. Therefore, ACE2 can regulate the tumor microenvironment and may serve as an immunotherapeutic target and a predictive biomarker of PD‐L1 blocking response.

**FIGURE 3:**
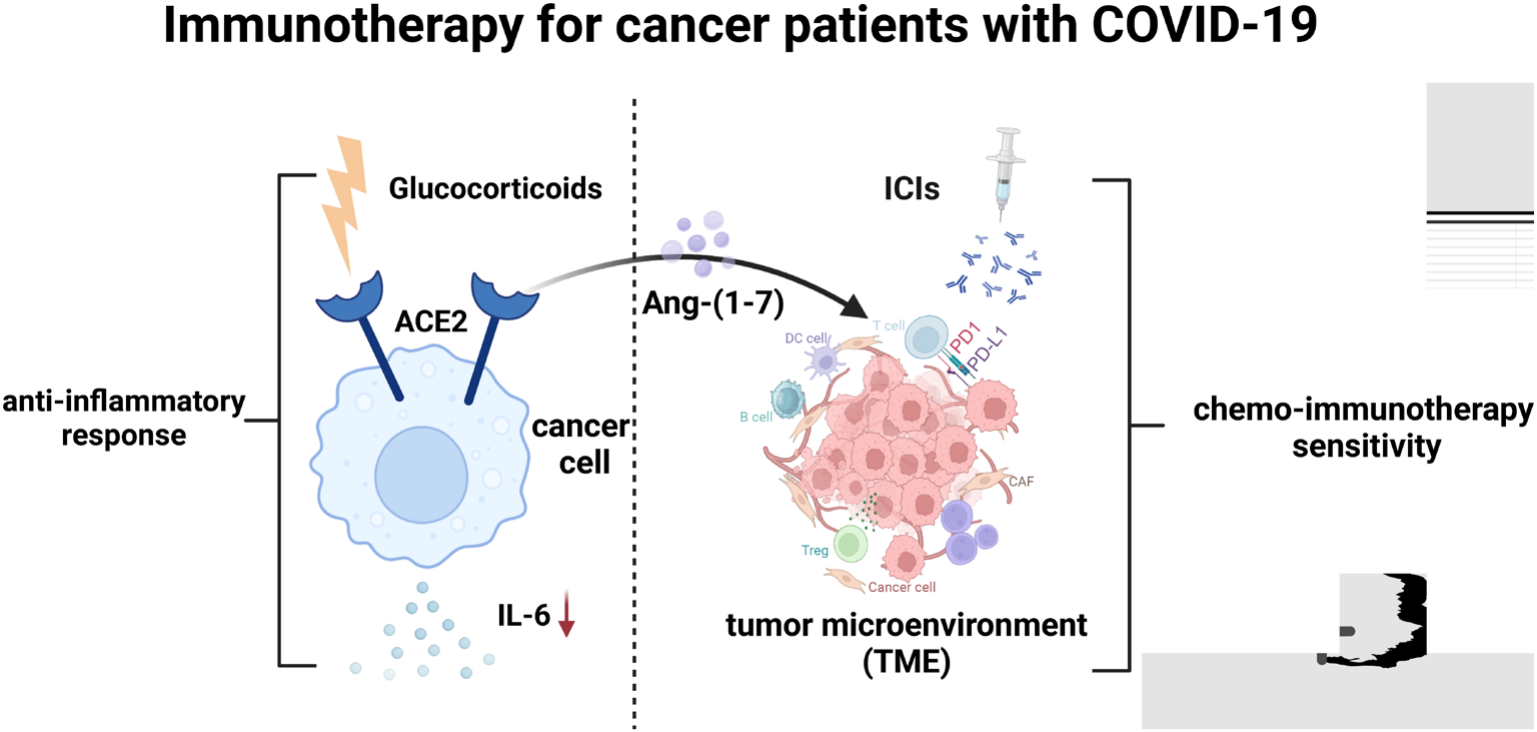
Immunotherapy for cancer patients with COVID‐19. Glucocorticoids can reduce the inflammatory response in cancer patients by activating ACE2 and reducing IL‐6 levels. ACE2 recognizes tumor cells. Ang‐(1–7), an enzyme product of AngII, increases the sensitivity of tumor cells to chemotherapy and immunotherapy by remodeling TME. The patient's harmful immune response may be reduced by blocking PD‐1/PD‐L1. Figure created with BioRender.com.

Xiang et al. found that glucocorticoids can enhance severe or critical COVID‐19 by activating ACE2 and reducing IL‐6 levels. They analyzed the effectiveness of multiple glucocorticoids, determining that hydrocortisone had the highest impact on ACE2 activation, followed in order by prednisolone, dexamethasone, and methylprednisolone [77]. In addition, after dexamethasone treatment, mortality was reduced in patients who developed COVID‐19 [80]. Synthetic GCs, such as dexamethasone and prednisolone, have long been used to treat a variety of diseases, including cancer, because of their antiinflammatory properties. However, the beneficial role of GC in epithelial cell cancers has been debated, and in colon, kidney, bladder, and prostate cancers, GC therapy can induce resistance to cytotoxic drugs or radiation therapy [81](Figure 3). JAK inhibitors, such as Ruxolitinib, can reduce the production of downstream inflammatory factors and inhibit virus‐induced immune overactivation by blocking the JAK–STAT signaling pathway. This has an important impact on improving the treatment of COVID‐19 patients and preventing serious complications [82, 83].

### 5.3 Treatment Considerations for Cancer Patients

Cancer patients often exhibit immunosuppression due to the disease itself or as a result of anticancer therapies such as chemotherapy and radiotherapy [62]. This immunosuppressed state can heighten susceptibility to SARS‐CoV‐2 infection and negatively impact the progression of COVID‐19. Chen et al. emphasized that immunosuppression, compounded by lymphopenia induced by chemotherapy, may lead to severe infections and complications [84]. For patients undergoing chemotherapy, adjustments such as dose reduction or increased intervals between cycles may reduce immunosuppression without significantly compromising efficacy [12]. The use of granulocyte colony‐stimulating factors (G‐CSF) can also be considered to mitigate chemotherapy‐induced neutropenia [13]. For those receiving immunotherapy, the impact on COVID‐19 outcomes is less clear, and decisions should be individualized based on the risk–benefit ratio. Moreover, cancer is associated with a hypercoagulable state, increasing the risk of thromboembolic events [85]. SARS‐CoV‐2 infection further exacerbates this risk due to virus‐induced endothelial dysfunction and inflammatory responses leading to coagulopathy. It is reported that COVID‐19 patients with cancer had a higher incidence of thrombotic complications compared to noncancer patients [86]. This prothrombotic status necessitates vigilant monitoring and potential prophylactic anticoagulation to mitigate the risk of venous thromboembolism. Building upon the therapeutic considerations, future research should focus on clinical evaluations of TMPRSS2 inhibitors, ADTs, and ACE2 modulators in cancer patients. Personalized approaches considering the specific cancer type, patient hormonal status, and ACE2/TMPRSS2 expression profiles will be critical for optimizing therapeutic outcomes. Integrating antiviral strategies with cancer treatments may offer a comprehensive approach to improve survival and quality of life for cancer patients with COVID‐19.

## 6 Conclusion

The insights gained from the analysis of TMPRSS2 and ACE2 in the context of COVID‐19 and cancer have significant clinical implications. First, the identification of TMPRSS2 as a prognostic biomarker in PCa and LUAD emphasizes the need for personalized treatment strategies that consider TMPRSS2 expression levels. For instance, patients with high TMPRSS2 expression in PCa may benefit from therapies that enhance immune infiltration, while those with low TMPRSS2 levels in LUAD might require alternative approaches to improve treatment responses.

Furthermore, the dual role of TMPRSS2 as both a facilitator of viral entry and a tumor suppressor necessitates careful consideration when developing therapeutic interventions. Inhibitors targeting TMPRSS2 could potentially reduce SARS‐CoV‐2 infectivity; however, their use must be balanced against the risk of promoting tumor progression in cancers where TMPRSS2 acts as a suppressor [87]. Additionally, the modulation of ACE2 expression presents a promising avenue for therapeutic intervention. Strategies aimed at optimizing ACE2 levels could enhance protective immune responses while mitigating the risk of severe COVID‐19 outcomes in cancer patients. In conclusion, the findings highlight the critical need for multidisciplinary approaches in managing cancer patients during the COVID‐19 pandemic. Clinicians should prioritize individualized risk assessments, considering the unique expression profiles of TMPRSS2 and ACE2 in each patient. This tailored approach will be essential for optimizing treatment outcomes and improving the overall management of cancer patients in the context of ongoing viral threats.

These findings highlight the critical need for personalized therapeutic strategies that consider the unique molecular profiles of ACE2 and TMPRSS2 expression in cancer patients. Such tailored approaches are essential for optimizing treatment outcomes and improving the management of cancer patients during the COVID‐19 pandemic.

## Author Contributions

The authors take full responsibility for this article.

## Ethics Statement

The authors have nothing to report.

## Consent

All authors have approved its publication.

## Conflicts of Interest

The authors declare no conflicts of interest.

## Data Availability

Data sharing is not applicable to this article as no new data were created or analyzed in this study.
